# GLP−1 receptor agonists for the treatment of obesity: Role as a promising approach

**DOI:** 10.3389/fendo.2023.1085799

**Published:** 2023-02-01

**Authors:** Jing-Yue Wang, Quan-Wei Wang, Xin-Yu Yang, Wei Yang, Dong-Rui Li, Jing-Yu Jin, Hui-Cong Zhang, Xian-Feng Zhang

**Affiliations:** ^1^ Department of Cardiovascular Medicine, The First Hospital of Jilin University, Changchun, China; ^2^ Department of Neurosurgery, The First Hospital of Jilin University, Changchun, China

**Keywords:** GLP-1R agonists, obesity, weight-reducing drugs, metabolic diseases, dual agonism

## Abstract

Obesity is a complex disease characterized by excessive fat accumulation which is caused by genetic, environmental and other factors. In recent years, there has been an increase in the morbidity, disability rate,and mortality due to obesity, making it great threat to people’s health and lives, and increasing public health care expenses. Evidence from previous studies show that weight loss can significantly reduce the risk of obesity-related complications and chronic diseases. Diet control, moderate exercise, behavior modification programs, bariatric surgery and prescription drug treatment are the major interventions used to help people lose weight. Among them, anti-obesity drugs have high compliance rates and cause noticeable short-term effects in reducing obese levels. However, given the safety or effectiveness concerns of anti-obesity drugs, many of the currently used drugs have limited clinical use. Glucagon-like peptide-1 receptor (GLP-1R) agonists are a group of drugs that targets incretin hormone action, and its receptors are widely distributed in nerves, islets, heart, lung, skin, and other organs. Several animal experiments and clinical trials have demonstrated that GLP-1R agonists are more effective in treating or preventing obesity. Therefore, GLP-1R agonists are promising agents for the treatment of obese individuals. This review describes evidence from previous research on the effects of GLP-1R agonists on obesity. We anticipate that this review will generate data that will help biomedical researchers or clinical workers develop obesity treatments based on GLP-1R agonists.

## 1 Introduction

In the past decade, obesity and related co-morbidities have become critical public health problems and serious medical conditions worldwide (1). A state of positive energy balance exists in the duration of the energy intake exceeding energy consumption, resulting in the storage of the excess calories in adipose tissue, which first leads to body phenotypic overweight (body mass index, BMI 25-30 kg/m^2^). It then develops into a weight disorder called obesity, defined as a BMI ≥30 kg/m^2^ (2). In pathology classification, there is no clear consensus on terminology. The most important sub-groups reviewed include abnormal metabolic obesity, normal metabolic obesity, abnormal metabolic weight, normal metabolic weight, and sarcopenic obesity (3). In Europe, the incidence of obesity is five times higher than it was after World War II, and the number of obese people doubles yearly (4). A study of BRICS (Brazil, Russia, India, China, and South Africa) countries during 2007-2010 found that obesity was associated with hypertension, angina, diabetes, and arthritis (5). Obesity is not just a risk factor for other diseases, such as cardiovascular diseases, type 2 diabetes (T2DM), arthritis, and some cancers (6), but also a complex disease with multiple causes, which has its own disabling ability, pathophysiology, and co-morbidities (7). Obesity dramatically increases the risk of diseases and disables ability. Herein, it is a significant health challenge that leads to a decline in life quality and life expectancy. So far, in the long run, obesity prevention and treatment strategies-whether at the individual or group level, have not been very successful (8). Lifestyle and behavioral interventions aimed at reducing calorie intake and increasing energy expenditure have limited effects because complex and lasting hormonal, metabolic and neurochemical adaptations can prevent weight loss and promote weight recovery (8). A systematic review and meta-analysis proved that weight loss had been shown to prevent and mitigate obesity-related complications (9). Weight-loss surgery effectively reduces weight and complications in patients with severe obesity; however, weight gain is a common complication following surgery (10). An intragastric balloon (IGB) is a usually safe, reversible, and less invasive way to cause weight loss based on occupying stomach space to increase satiety. However, regarding weight loss, the results of using IGBs cannot be compared with bariatric surgery (11). Drug therapy for weight loss has made significant progress. However, many anti-obesity agents that have entered human clinical trials have shown unacceptable adverse events; many of them cannot be used for more than 3 months; the curative effect is moderate and suboptimal in the long term (12). The leptin-melanocortin axis, the opioid system, glucagon-like peptide-1 (GLP-1)/glucagon-like peptide-1 receptor (GLP-1R) system, and fibroblast growth factor 21 (FGF21)/its receptor complex FGFR1c/β-klotho axis are several common pathways confirmed by researches and pharmacological targets that play an essential role in regulating energy balance and feeding behavior (2). GLP-1 analogs and GLP-1R agonists can exert hypoglycemic effects and reduce weight. However, natural GLP-1 can easily be degraded by dipeptidyl peptidase 4 (DPP-4) *in vivo* and lose its activity. In order to make GLP-1 better used in the clinic, drug developers modified its structure and developed a series of GLP-1R agonists (13). Many clinical trials have proven GLP-1R agonists’ effectiveness and safety in treating or preventing obesity (14).

**Scheme 1:**
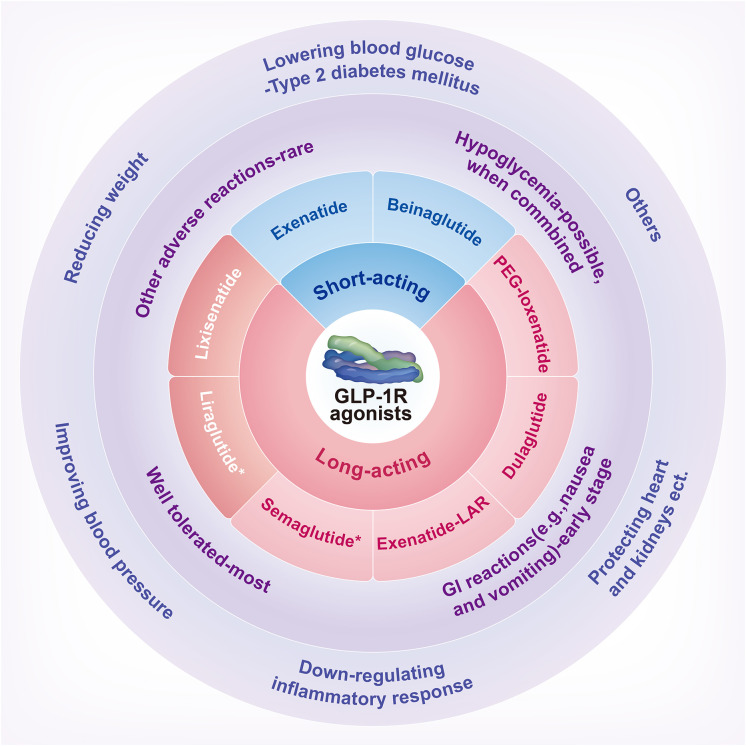
A schematic overview of GLP-1 receptor agonists: classification, drug safety, and effectiveness. GI, Gastrointestinal; GLP-1R, Glucagon-like peptide-1 receptor; PEG, Polyethylene glycol. *Liraglutide and Semaglutide have been approved by the United States Food and Drug Administration for weight loss.

**Figure 1:**
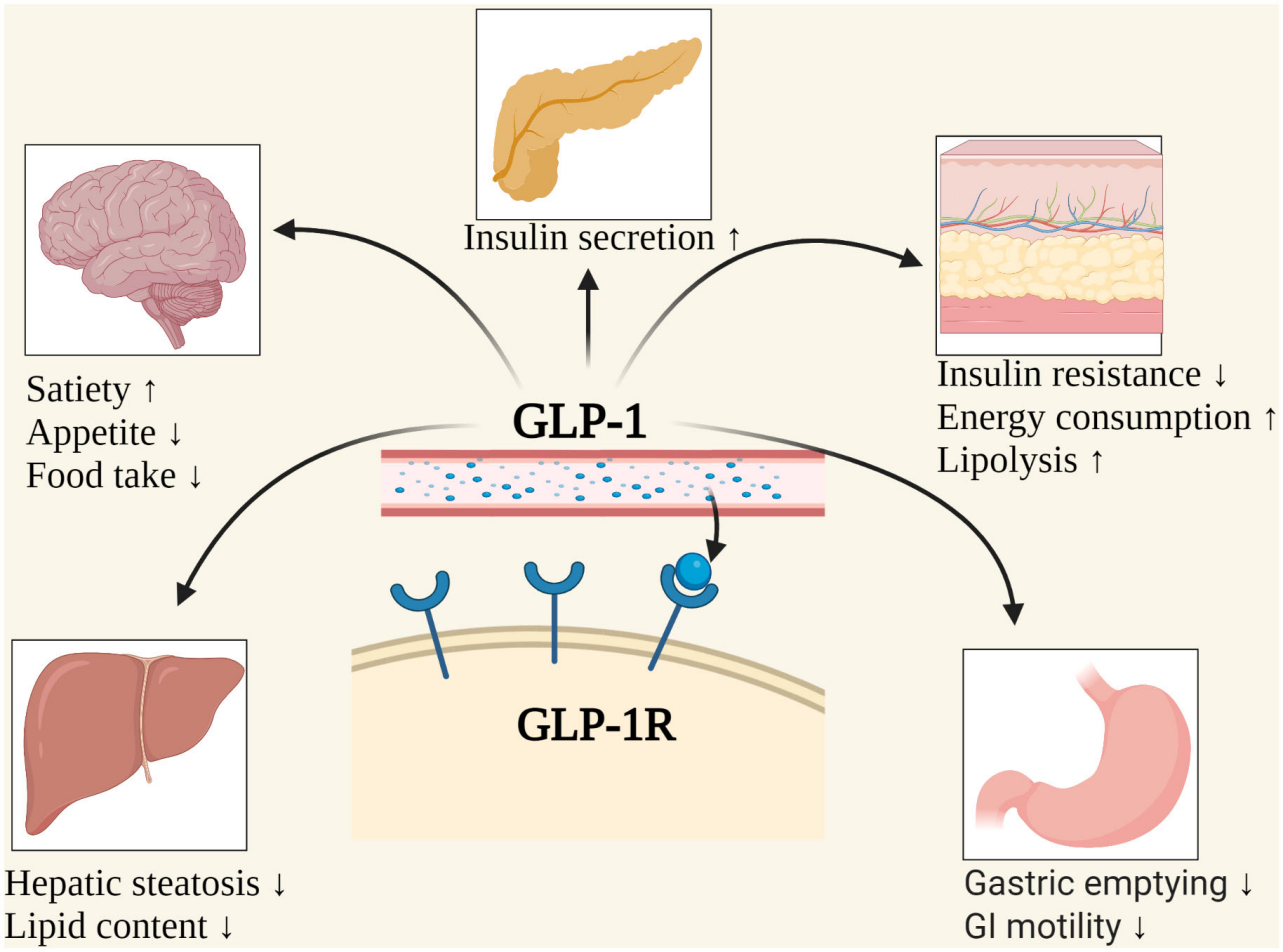
The possible mechanisms of weight loss caused by GLP-1. GI, Gastrointestinal; GLP-1, Glucagon-like peptide-1; GLP-1R, Glucagon-like peptide-1 receptor. Created with BioRender.com.

GLP-1, a 30- or 31-amino-acid-long peptide hormone, is a gastrointestinal peptide secreted by the intestinal tract that potentiates insulin release and reduces glucagon’s concentration in physiological conditions (15, 16). In 1983, it was found to be a cleavage product of proglucagon processing and secreted by intestinal epithelial endocrine L cells (17). It also comes from α-cells in the pancreatic islet and neurons in the nucleus of the solitary tract (18). GLP-1 is necessary for standard glucose tolerance and functions through specific GLP-1Rs, which belong to the G-protein-coupled glucagon receptor family, expressed in islet β-cells and stomach, small intestine, mucosa, heart, and other cell types (18, 19). GLP-1R agonists are a group of drugs based on the entero-insular axis (19). D’Alessio et al. (18) demonstrated that circulating peptides mediate insulinotropic activity with either bariatric surgery or treatment with long-acting GLP-1R agonists. Therefore, GLP-1R agonists may be new and promising treatment alternatives for obese subjects. However, the mechanism of GLP-1R in treating obesity is still uncertain, and more clinical evidence is needed for its long-term safety and efficacy. A schematic overview of GLP-1R agonists is summarized in **Scheme 1**. The possible mechanisms of weight loss caused by GLP-1R agonists is shown in **Figure 1**. Comparisons of common weight loss drugs are made in **Table 1**.

**Table 1:** Anti-obesity drugs: principal categories, functions, side effects, half-life and usage (20, 21).

Major Categories	Functions	Primary side effects&Limits	Half-life/hours	Usage
Orlistat	•Weight loss •Reducing the risk of regaining weight already lost •Only in adults that are overweight or obese	•Side effects Allergic; Pain in the stomach or lower back; Kidney and liver problems.	1-2	120mg, TID (P.O.)/60mg, TID(P.O.)
Naltrexone-Bupropion	•Bupropion Antidepressant; Decreasing appetite. •Naltrexone Blocking the effects of opioids/alcohol; Curbing hunger and food cravings. • Contrave Managing weight in obese/overweight adults.	•Side effects Allergic; Seizures; Eating disorder; High blood pressure; Suicidal thoughts or actions. •Limits of use Effect on cardiovascular morbidity and mortality: unclear; Safety and effectiveness of combined medication: unclear.	21/5	8/90 mg,BID (P.O.)
Phentermine-Topiramate^*^	•BMI≥ 30 kg/m^2^ •BMI≥ 27 kg/m^2^ with hyT2DM/dyslipidemia etc	• Side effects Allergic; Mood changes; Trouble sleeping; Increases of acid in the blood; Kidney stones. •Limits of use Effect on cardiovascular morbidity and mortality: unclear; Safety and effectiveness of combined medication: unclear.	20/65	•Start treatment with 3.75/23mg, QD (P.O.) •Recommended dose of 7.5/46mg, QD (P.O.) •Dose escalation to 15/92mg, QD (P.O.)
Lorcaserin	•BMI≥ 30 kg/m^2^ •BMI≥ 27 kg/m^2^ with hypertension/T2DM/dyslipidemia etc	•Side effects Anxiety; Bladder pain; Bloody or cloudy urine; Blurred vision; Body aches or pain; Cancer.	11	10mg, BID(P.O.)
Semaglutide	• T2DM •T2DM and established cardiovascular disease •Obesity	•Side effects Bladder pain; Bloody or cloudy urine; Chills; Cough; Diarrhea etc. •Limits of use Patients with a history of pancreatitis: unclear; Patients with T1DM: unclear.	165	2.4 mg,QW(Subcut)
Liraglutide	•T2DM •T2DM and established cardiovascular disease •Obesity	•Side effects Bladder pain; Bloody or cloudy urine; Chills; Cough; Diarrhea etc. •Limits of use Patients with a history of pancreatitis: unclear; Patients with T1DM: unclear.	13	3mg,QD(Subcut)

QD, One time a day; QW, One time a week; BID, Two times a day; I.V., Intravenous; TID, Three times a day; P.O., By mouth; BMI, Body mass index; T2DM, Type 2 diabetes mellitus; T1DM, Type 1 diabetes mellitus; Subcut, Subcutaneous injection. *The anti-obesity drugs' estimated percentile ranking of weight loss of 10% in participants who took weight-loss medications for more than a year is Phentermine-Topiramate, 54%; Liraglutide, 34%; Naltrexone-Bupropion, 30%; Lorcaserin, 25%; Orlistat, 20% (20).

## 2 Relationship between GLP-1 and obesity

The mechanism for the decrease in GLP-1 secretion is incompletely understood in obese people. Ranganath (22) et al. believe that the decrease of GLP-1 in obese subjects may be related to increased plasma non-esterified fatty acids (NEFA). Two clinical studies in simple obesity patients found that higher fasting and postprandial NEFA levels, the insulin-promoting effect of GLP-1 and its postulated role as a satiety factor may inhibit nutrition-mediated secretion (23).

In the central nervous system, proglucagon and proglucagon-derived peptides (GLP-1, glucagon-like peptide-2, GLP-2, and oxyntomodulin, OXM) are only present in a small group of neurons in the nucleus tractus solitarius (24, 25). These constitute the neural pathway that connects the “visceral sensory” brainstem and the hypothalamic nucleus to participate in energy homeostasis. Experiments on mice have shown that intraventricular administration of GLP-1, GLP-2, and OXM could significantly reduce food intake (25, 26). Central GLP-1 activation may be combined with GLP-2 and/or OXM activation to form a potential pharmacological tool to reduce meal intake and possibly increase energy consumption (25, 27). The expression of GLP-1 and/or GLP-1R was found in hypothalamic feeding centers (28, 29), hindbrain matrix (30), the lateral parabrachial nucleus of the posterior nucleus (31), and mesolimbic substrates (32–34), etc. parts which directly effect on appetite and lead to weight loss.

In addition, the GLP-1 can inhibit postprandial gastric emptying and reduce gastric acid secretion, inhibit gastric and duodenal peristalsis by inhibiting vagus nerve, and increase the pressure of pylorus, thus reducing appetite, causing a reduction in body weight and participating in the so-called “ileal braking” effect (19, 35). Beiroa (36) et al. injected GLP-1 analog, liraglutide (LIR), into mice. LIR was found to stimulate the thermogenesis of brown adipose tissue and the browning of adipose cells, independent of the intake of nutrients. It can increase the thermogenesis and utilization of triacylglycerol-derived fatty acids and glucose in brown adipocytes and reduce the lipid content through the central GLP-1R agonists’ signaling pathway, such as central AMP-activated protein kinase or sirtuin-1 (SIRT1), also known as NAD-dependent deacetylase SIRT1 (36–39).

## 3 Experimental evidence of GLP-1R agonists

GLP-1R agonists can be divided into short-acting and long-acting preparations according to the time-effect and the volume of injections. Short-acting preparations include beinaglutide (BN) and exenatide (EX), which generally need to be injected 2-3 times a day. Long-acting preparations include lixisenatide (LIXI) and LIR, which are injected once a day. Long-acting preparations also include semaglutide (SMG), dulaglutide (DUL), long-acting release formulation of exenatide (EX-LAR), and polyethylene glycol loxenatide (PEX-168), which generally need to be injected once a week. The data in this section is from animal and human trials.

### 3.1 Short−acting GLP−1 receptor agonists

#### 3.1.1 Beinaglutide

BN is a recombinant human GLP-1(7-36) acid, obtained by genetic engineering technology, and the amino acid sequence of its active component is the same as that of GLP-1 in the human body. As the first original research drug in the field of diabetes in China, it has been recommended for meal injection and approved for the hypoglycemic intervention of T2DM (40), especially for patients with poor blood glucose control by metformin alone. BN can also be used for patients with poor blood glucose control alone. The starting dose is 0.1 mg (50 µl) three times daily and is injected subcutaneously 5 minutes before meals. After 2 weeks of treatment, the dose should be increased to 0.2 mg (100 µl) three times daily. This product has a half-life of about 11 minutes and can effectively control blood glucose within 2 hours after the meal. The drug is quickly eliminated in the body without accumulation.

In clinical studies, Wang (41) et al. included 36 patients (T2DM) with BMI ≥ 24 kg/m^2^. After treatment with BN for 3 months, BMI, fasting blood glucose, glycosylated hemoglobin, visceral and subcutaneous fat, silk protein E1, leptin, C-reactive protein (CRP), and tumor necrosis factor decreased significantly; the level of CRP is related to the weight loss percentage treated with BN. A real-world study from China evaluated the actual efficacy of BN in treating overweight/obese T2DM patients in an actual diagnosis and treatment environment. The study found that the average weight decreased by 10.05 kg after 3 months of treatment (40). In animal studies, Fang (42) et al. studied the pharmacological and pharmacokinetic efficacy of BN in C57BL/6 and ob/ob mice. Results showed that BN could reduce glucose levels, improve insulin secretion in glucose tolerance tests in a dose-dependent manner, and inhibit food intake and gastric emptying. BN inhibited meal intake at higher doses for more than 4 hours, leading to weight loss in ob/ob mice after about two weeks of treatment. In the nonalcoholic steatohepatitis (NASH) model, BN can reduce liver weight and steatosis and improve insulin sensitivity. There were significant changes in fatty acid β-oxidation (Ppara, Acadl, Acox1), mitochondrial function (Mfn1, Mfn2), antioxidation (Sod2), SIRT1. This suggests that BN may be an effective treatment for obesity and NASH (42). A clinical study involving 78 non-diabetic overweight/obese patients found that BN may be a more effective treatment option for overweight/obese Chinese than metformin (43). For example, a patient with a BMI of 17.96 kg/m^2^ found that BN did not make him lose weight but increased it. The researcher believes that it may be related to the patient’s habit of excessive intake (44). An animal experiment on mice found that the mechanism of BN treating obesity is targeting the composition of major lipid classes and the expression of genes in lipid metabolism in adipose tissue to counteract high-fat diet-induced obesity (45).

BN can reduce body weight, inhibit inflammatory reactions, directly be excreted by the kidney, and have fewer adverse reactions. However, relevant research on it has not been found abroad. In the future, more prospective randomized controlled clinical studies can be conducted for patients with diabetic obesity or simple obesity.

#### 3.1.2 Exenatide

As the first GLP-1R agonist approved for T2DM, EX is a new class of compound and a synthetic product of exendin-4, which is a compound naturally derived from the Gila monster and has multiple hypoglycemic mechanisms: inhibiting of glucagon release from α cells, slowing of gastric emptying, decreasing appetite and increasing glucose-dependent insulin secretion (12, 46, 47). It is similar to human GLP-1 in function (46). Human GLP-1 was rapidly degraded by DPP-4, while EX showed prolonged kinetics because of its resistance to proteolysis of DPP-4 (48). EX has a relatively short half-life of 2.4 hours, which can be detected in plasma within 15 minutes after administration, and can still be detected 15 hours after a single subcutaneous injection > 0.2 µg/kg (12).

Lipotoxicity induced by saturated free fatty acids plays a vital role in renal injury in obese patients (49). It is found that EX can reverse the damage of renal tubular epithelial cells caused by a high-fat diet, and its effect is applied to simvastatin by reversing the down-regulation of SIRT1, preventing the production of reactive oxygen species and inhibiting the apoptosis of mitochondria (50).

There is little research on the weight loss effect of EX on simple obesity patients, and more clinical trials are conducted on patients with T2DM. A randomized, double-blind, placebo-controlled, parallel-group study showed that among the patients with T2DM treated with EX monotherapy, EX could improve glycated hemoglobin, fasting blood glucose, and postprandial blood glucose control, reduce weight, improve β cell function, and adjust blood pressure. EX is well tolerated (51). In a triple-blind, placebo-controlled 30-week clinical study involving patients with T2DM with a BMI of 33 ± 6 kg/m^2^, it was found that the weight loss in the EX treatment group was significantly higher than that in the control group. The primary adverse reaction to EX is nausea, suggesting that nausea may be the primary mechanism of weight loss (52). A 24-week multicenter, randomized, double-blind, placebo-controlled clinical study conducted by Apovian (53) found that EX combined with lifestyle adjustment could significantly reduce the weight of patients, all of which were 6.16 ± 0.54 kg. However, the incidence of nausea was higher than that of the control group. At present, the studies of EX on non-diabetic obesity mainly include 152 cases of simple obesity (weight loss of 5.1 kg) (54), 60 cases of polycystic ovary syndrome (weight loss of 3.2-6.0 kg) (55), and 10 cases of metabolic syndrome (weight loss of 3.7 kg) (56), among which the most common adverse reaction is still vomiting.

To sum up, EX can reduce the weight of diabetic patients with or without obesity, and it can also reduce the weight of simple obesity and polycystic ovary syndrome/metabolic syndrome with obesity. Clinical trials have shown that it has the potential to cause weight loss in non-diabetic obese individuals (57). The disadvantage is that it can cause vomiting; injection therapy is not convenient. Thus, more prospective clinical studies on non-diabetic obese patients are needed.

### 3.2 Long−acting GLP−1 receptor agonists: once-daily

#### 3.2.1 Lixisenatide

LIXI is a once-daily GLP-1R agonist. As a synthetic analog of its endogenous exendin-4, compared with exendin-4, LIXI deleted one proline and added six lysine residues, which increased its binding affinity with the GLP-1R by four times and increased its circulating half-life (58, 59). Furthermore, the dissociation rate with the receptor is slow, and the high affinity and slow dissociation with the receptor prolongs the time of pharmacological effect (58, 60). The indication is to effectively reduce glycosylated hemoglobin levels in patients with T2DM by reducing fasting and postprandial blood glucose levels (61, 62). LIXI is administered subcutaneously, quickly absorbed into the blood, without biotransformation in the liver, and is degraded by proteolytic enzyme and then eliminated by the kidney (63, 64). Along with its role in lowering blood glucose levels, LIXI has many other functions, such as cardiovascular benefits, delaying gastric emptying, protective effect on pancreatic beta-cells, and increased insulin mRNA expression and hormone secretion (65). Also, LIXI is well tolerated, and nausea and vomiting are the most commonly reported adverse events (66, 67).

A prospective clinical study from Spain found that besides controlling blood glucose (fasting blood glucose and glycosylated hemoglobin), LIXI can also improve blood lipids, especially total cholesterol and triglyceride (68). The exact mechanism may be that LIXI reduces chylomicron triacylglycerol by delaying gastric emptying by increased clearance (69). In iGlarLixi (insulin glargine: LIXI = 1:1) study, adult Japanese patients with T2DM lost more weight compared to the iGlar (insulin glargine U100) study (-0.51 kg vs. +0.55 kg). However, iGlarLixi patients had more gastrointestinal-related adverse, primarily nausea (16.9% vs. 0.8%) in this 26-week, randomized, open-label study (70). At the same time, the research of GetGoal-Duo1 and GetGoal-L also found that when the basic insulin treatment was not effective, the combination of LIXI could not only significantly improve fasting blood glucose and glycosylated hemoglobin but also significantly reduce weight, with the weight dropping by 0.9 kg and 1.3 kg respectively (71). In GetGoal-S, a subanalysis of GetGoal-S and GetGoal-F1 studies, they found that LIXI could significantly reduce subjects’ body weight (− 1.76 ± 0.20 kg;-1.12 kg; -2.6/2.7 kg), respectively (72–74). The main adverse events of LIXI are gastrointestinal adverse reactions, mainly manifested as nausea and vomiting in the initial stage (75, 76). Compared with EX, the frequency of subcutaneous injection of LIXI is less, and the incidence of gastrointestinal adverse reactions and hypoglycemia is lower. Compared with LIXI, LIR is more effective in improving blood glucose and reducing weight, and both treatments are well tolerated (75, 77, 78).

In clinical studies, LIXI has shown the benefits of weight loss, which is well tolerated by patients and can meet the needs of overweight patients for weight control. Given that there are no major, obvious differences in tolerability, efficacy on glycemic parameters, weight loss, or overt advantages regarding administration, it is likely that the use of LIXI in place of other GLP-1 agonists will be based on cost (79).

#### 3.2.2 Liraglutide

LIR was formed by adding a 16-carbon palmitoyl fatty acid to the 26th position of GLP-1 by gene recombination technology and replacing the 34th position of lysine on GLP-1 with arginine. These structural modifications increase aggregation and promote non-covalent binding of the albumin, thus inhibiting the DPP-4, which is injected subcutaneously in the form of isotonic fluid. LIR is suitable to be administered once a day, with a t_max_ of 9~13 hours and t_1/2_ of 13 hours (80–82). Clinically, it is mainly used to treat T2DM and causes weight reduction when used in large doses (3mg/day) (83). The United States Food and Drug Administration (FDA) approved it to treat obesity. Its main principles include that it can inhibit the feeding center, thus reducing appetite; it can delay gastric emptying, resulting in slow digestion and absorption of food, to achieve the role of diet control (84–86).

Compared with the treatment of short-acting GLP-1R agonists, LIR has a greater improvement in lowering glycated hemoglobin and fasting blood glucose. It has fewer side effects than EX twice a day (87). A clinical study involving 564 adults (BMI 30-40 kg/m2, without T2DM) found that compared with orlistat (3×120 mg, weight loss of 4.1 kg), LIR (subcutaneous administration of 1.2 mg/d, 1.8 mg/d, 2.4 mg/d or 3 mg/d) had an average weight loss of 4.8-7.2 kg, the two-year extended experiment of which also showed similar results (88, 89). The relevant study also found that the 1-year average weight loss of participants (LIR 3.0 mg) was 9.2 kg reporting nausea/vomiting episodes, while that of participants without nausea/vomiting was 6.3 kg. Researchers believe that nausea/vomiting is related to greater weight loss (90). A three-year obesity and prediabetes trial found that LIR 3 mg can improve health benefits in reducing the diabetes risk of patients with obesity and prediabetes (91). Pi-Sunyer (92) et al. enrolled 3731 patients without T2DM (BMI ≥ 27 kg/m^2^ or 30 kg/m^2^) and gave LIR 3mg daily subcutaneous injection. After 56 weeks of treatment, the average weight loss of the patients was 8.40 ± 7.3 kg, and the most common adverse reactions were mild or moderate nausea and diarrhea. A 56-week trial included 422 patients with BMI≥30 kg/m^2^ or BMI≥27 kg/m^2^ with dyslipidemia and/or hypertension (non-diabetic). These patients reduced their initial weight by ≥5% through a low-calorie diet during the 4-12 weeks pre-trial induction period. It was found that more patients in the LIR group than the placebo group maintained their weight loss by ≥5%, which indicated that LIR 3.0 mg/d is expected to improve the maintenance of weight loss (93).

GLP-1 analog has 97% homology with human GLP-1 and has been approved for the clinical treatment of diabetes and obesity. However, it should be noted that combined with insulin may lead to hypoglycemia (94). It is worth mentioning that China's FDA said that the application for a marketing license for LIR injection for obesity or overweight indications had been accepted, which was the first time that the weight control indications of GLP-1R agonists have been accepted in China.

### 3.3 Long-acting GLP−1 receptor agonists: Once-weekly

#### 3.3.1 Semaglutide

SMG is an analog of long-acting GLP-1, mainly used to control T2DM, and is also suitable for promoting weight loss in people with mild obesity (95, 96). The usage for treating obesity is subcutaneous injection once a week. For both diabetic and non-diabetic patients, the drug can reduce weight (97, 98). Here, we mainly summarize the relevant experiments on the SMG treatment effect in people with obesity (STEP) programs.

More than 1,200 patients with T2DM complicated with obesity were randomly divided into 1mg SMG group, 2.4mg SMG group, or placebo group and given once a week in STEP2 research. The weight loss ranges of the two treatment groups were -6.9 kg (-7%) and -9.7 kg (-9.6%), respectively. The 2.4 mg group lost the most weight, which means that among adults suffering from overweight or obesity and T2DM, SMG 2.4 mg once a week has achieved significant clinically significant weight loss (99). In the STEP1 trial (100), Wilding et al. recruited 1961 non-diabetic adults with a BMI of 30 kg/m^2^ (or ≥27 kg/m^2^ with more than 1 body weight-related coexisting disease). The SMG group (once 2.4 mg, once a week) was treated for 68 weeks, and the average weight loss in the SMG group was 15.3 kg. More patients in the SMG group achieved a weight loss of ≥ 5% (86.4% vs. 31.5%), ≥ 10% (69.1% vs. 12.0%), and ≥ 15% (50.5% vs. 4.9%), compared with the placebo group. STEP 8 and STEP 3 showed similar results (101, 102).

In the United States, oral and injectable forms of SMG are approved for treating T2DM, while only injectable forms are approved for treating obesity (103). Like other GLP-1R agonists, SMG often causes adverse reactions. The main adverse reactions were gastrointestinal side effects, including nausea, diarrhea, and vomiting. These adverse reactions are generally mild to moderate and gradually alleviated in most patients (100, 104). Like LIR, the race of patients involved in related clinical studies is relatively small. More clinical trials aimed at obese people in Asia and Africa are expected to prove its effectiveness and safety. Regarding the treatment of obesity, we expect SMG to be approved by more countries’ FDA in the future.

#### 3.3.2 Dulaglutide

DUL is a long-acting GLP-1R agonist which can promote insulin secretion, inhibit gastric emptying, and reduce appetite. It is approved for treating hyperglycemia in patients with T2DM in many countries (105, 106). Like other GLP-1R agonists, when used alone or treated with non-insulin secretagogues, DUL treatment is associated with weight loss or decreased weight gain (107). It has a long half-life period and is injected once a week. The average peak time of subcutaneous injection is 48 hours. The most common adverse reactions were nausea, headache, vomiting, and diarrhea (108). Among them, gastrointestinal adverse reactions mainly occurred in the early stage of the first drug use, with mild to moderate degrees (109). Here, we mainly summarize the relevant research on assessing the weekly administration of DUL (AWARD).

AWARD-6 is a randomized, open, parallel-grouped, multicenter, phase 3, non-inferiority study comparing the safety and efficacy of weekly DUL and daily LIR in patients with uncontrolled T2DM treated with metformin. Compared with LIR once a day, once weekly subcutaneous injection of DUL similarly lowers body weight: –2.90 kg (-3.61 kg for LIR) after 26 weeks of treatment (109). At 26 weeks after treatment, the weight loss was -2.29 ± 0.24 kg for 1.5 mg DUL, -1.36 ± 0.24 kg for 0.75 mg DUL, and -2.22 ± 0.24 kg for metformin (110). In patients with T2DM who are poorly controlled by metformin, increasing DUL from 1.5mg to 3.0mg or 4.5mg can provide glycosylated hemoglobin and weight loss related to clinically relevant doses and has similar safety (111). It is worth mentioning that in the AWARD-2 study, there were several cases of pancreatitis caused by DUL (112). Therefore, during DUL treatment, we should pay close attention to the related signs of pancreatitis, and if pancreatitis occurs, we need to stop the drug immediately (113).

In summary, the recommended clinical treatment for diabetes is 0.75 mg or 1.5 mg. Studies have found that higher doses can further reduce glycosylated hemoglobin and body weight, and the safety is similar to low doses. However, DUL’s long-term efficacy and safety need to be further studied.

#### 3.3.3 Long-acting release formulation of exenatide (Microspheres)

Polymer materials commonly used in microsphere preparation are polylactic-co-glycolic acid (PLGA), which is approved by FDA and has good safety and biodegradability. It is often used as a drug carrier and biological scaffold. EX microspheres are a long-acting release of EX. After weekly subcutaneous injection, EX was slowly released from the microspheres by diffusion and microsphere rupture and reached a stable plasma concentration about 6-8 weeks after treatment (114). Nausea and vomiting are less frequent in patients receiving EX-LAR than in patients taking EX (114).

A 30-week randomized non-inferiority study comparing 2 mg once-weekly EX-LAR with 10 µg twice-daily EX in 295 patients with T2DM. In terms of weight loss, there was no significant difference between weight loss with EX (-3.6 kg) and with EX-LAR (-3.7 kg) (115). A study involving treatment up to 104 or 117 weeks in 134 patients with T2DM showed EX microspheres-naïve patients experienced weight reductions of -2.7 kg (116). Hirsch et al. (117) found that after 24 weeks of drug treatment of once-weekly EX-LAR or dapagliflozin or co-administered, nondiabetic women with a BMI (≥30 kg/m^2^ and ≤ 45 kg/m^2^) and polycystic ovary syndrome all lost weight. Among them, weight loss was the most in the double treatment.

Once-weekly treatment with EX-LAR (considering the potential beneficial effects of GLP-1R activation on β cells and weight loss associated with many patients) is a promising potential strategy for diabetes prevention. Similarly, once-weekly EX-LAR preparation can be used as an anti-obesity treatment in patients without T2DM or impaired glucose tolerance (118). EX long-acting preparation can reduce weight, but there are few related clinical studies, and most focus on patients with T2DM.

#### 3.3.4 Polyethylene glycol loxenatide

The early GLP-1R agonist has many defects, such as multiple injections and a high incidence of adverse reactions in the digestive tract, which limits its clinical application. PEX-168 is a new agent of long-acting GLP-1R agonist, which is molecularly modified from EX *via* amino acid modification and PEGylation (119), the first GLP-1R agonist modified by PEG to achieve long-term pharmacological action (120, 121). It has the advantages of a prolonged administration interval (once a week), few adverse reactions in the digestive tract (122), and adverse effects reported ranging from ‘mild’ to ‘moderate’ (122, 123).

Guo (120) et al. found that PEX-168 significantly reduced the body weight of simple obese mice. The weight loss of the low dose (0.03 mg/kg) group, medium dose (0.1 mg/kg) group, and high dose (0.33 mg/kg) group were about 2g, 4g, and 1g (8 weeks). A multicenter, randomized, double-blind, multiple dose-escalation study showed that after 4 weeks of treatment of PEX-168 with different doses, the weight decreased by -0.8-1.8kg. After 8 weeks of treatment, the weight decreased by -1.4-3.3kg (122).

PEX-168 is a China-domestic long-acting GLP-1R agonist approved by the China's FDA for marketing and used for blood glucose control of adult patients with T2DM.

## 4 Others: GIPR/GLP-1R dual agonists and GIPR/GLP-1R/GCGR triagonists

Intestinal endocrine K cells produce gastric inhibitory polypeptide (GIP). Like GLP-1, GIP stimulates insulin secretion from pancreatic β cells in a glucose-dependent manner (124). Glucagon (GCG) is a 29 amino acid peptide secreted by pancreatic α cells when blood glucose levels are low. Its ability to increase energy expenditure has been known for over 60 years (125). Recently, several multi-targeting agonists of GIPR, GLP-1R, or GCGR for treating T2DM and obesity have been in clinical trials. These agonists are developed to maximize metabolic benefits and reduce side effects (126). Therefore, the research and development of the relevant drugs and the replacement therapy of double GIP-GLP-1R agonists (e.g., tirzepatide) and triple GIP-GCG-GLP-1R (e.g., peptide 20) agonist are key research fields.

Research on obese mice suggests that unimolecular poly-pharmacology is an effective means to deal with various mechanisms leading to obesity and further indicates that GCGR activation is the distinguishing factor between single or double agonists and triple agonists of incretin receptors (127). Studies in knock-out mice confirms the significant potential for novel triple-acting hybrid peptides as therapeutic options for obesity (128). Five clinical trials (SURPASS 1-5 trials) conducted in patients with T2DM showed that tirzepatide at 5-15 mg per week could reduce body weight (5.4-11.7kg), which was never seen with a single drug (129). Karagiannis et al. found that tezepatide had a significant dose-dependent advantage in weight loss compared with placebo, GLP-1R agonists, and basal insulin (130). Zhao et al. provide valuable insights into the structural basis of the functional versatility of tirzepatide and peptide 20 by cryo-electron microscopy (126). Researchers interested in this direction can see more details on the efficacy of GLP-1R/GCGR agonists in the treatment of obesity in Sanchez-Garrido’s review (131).

GLP-1R agonists improve glucose homeostasis in patients with T2DM, cause weight loss, and benefit cardiovascular health over time. However, dose-dependent gastrointestinal effects limit efficacy, so drugs with GLP-1 and GCG pharmacology can also target alternative pathways and may expand the therapeutic index (132).

## 5 Future research outlook

Over a relatively short period, obesity is a significant public health problem rising. Despite numerous studies, the causes of the obesity epidemic are still not fully understood, and traditional calorie-restricted diets still lack long-term effects (133). It needs effective prevention and treatment. Weight loss surgery may be the most effective treatment for morbid obesity, but we need less radical choices, such as drugs (134). However, obesity is a global health challenge with few drug options (100). The long-term weight-loss agents approved by the FDA of the United States are orlistat, lorcaserin, naltrexone-bupropion, topiramate phenylacetate, LIR, SMG etc. (20). Orlistat is the only weight-reducing drug approved for marketing in China so far. However, these diet pills are controversial because of their safety and long-term efficacy (135, 136). In 2005, a new treatment of T2DM based on the action of GLP-1 was introduced (137). Two GLP-1R agonists have been approved in the United States to treat obesity: semaglutide and LIR, both of which are injected subcutaneously. Whether the patient has diabetes or not, these drugs should be the first-line drugs for obesity (138). For patients with diabetes, the side effects, injection demand, and cost of these drugs should be weighed against the effects of improving blood glucose and losing weight. Currently, GLP-1R agonists are mainly targeted on the endogenous GLP-1R signaling system, which causes GLP-1R agonists to have many pharmacodynamic characteristics like GLP-1. These drugs vary, like changing pharmacokinetics, and different drugs have different dose plans for daily or weekly injections (139).

Besides lowering blood glucose and reducing weight (incredibly visceral fat), GLP-1R agonists can also lower blood pressure, improve blood lipid disorder, and reduce fatty liver (16, 140). The inhibitory effect of GLP-1 on gastric emptying is dose-dependent, and the use of lower doses of GLP-1 in patients with T2DM may also be suitable for blood glucose control (141). Also, GLP-1R agonists can protect the heart and kidneys, reduce the risk of cardiovascular events, and delay the progress of diabetic nephropathy, which is especially important for people with diabetes. The common adverse reactions of GLP-1R agonists include gastrointestinal reactions, mainly loss of appetite, nausea, vomiting, diarrhea, abdominal pain, etc., primarily mild or moderate. To alleviate the gastrointestinal reaction of patients, at the beginning of the application of these drugs, clinical workers should know that the dosage should be gradually increased from a small dosage to enhance patients’ tolerance. Generally, single use will not lead to hypoglycemia, but if combined with sulfonylureas or insulin, attention should be paid to prevent hypoglycemia (142). Other adverse reactions are rare, such as pancreatitis and rash.

Given that GLP-1R agonists have superior weight loss effects and good safety, some of its products have been officially approved by FDA for chronic weight management of obese or overweight adults. The GLP-1R agonists are promising candidates for the treatment of obesity. Currently, except for LIR and SMG, the numbers of related clinical studies of other GLP-1R agonists are few, especially in obese or overweight people without diabetes. Meanwhile, research on the mechanism and efficacy of GLP-1R/GIPR dual agonists and GLP-1R/GIPR/GCGR triple agonists paves the way to a ground-breaking therapy specific for obesity, which suggests multi-target drugs may have more advantages than a single target. Together, GLP-1R, GIPR, and GCGR agonists are expected to see more clinical trial evidence to prove their efficacy and safety in treating obese patients.

Pedrosa et al. (143) summed up GLP-1R agonists to treat obesity and prevent cardiovascular disease. Liu et al. (144) expounded on the modified GLP-1R agonists from the perspective of the patent. Jensterle et al. (145) presented the primary outcomes of clinical trial programs called SCALE and STEP and studies on the efficacy of GLP-1R agonists in pediatric obesity. In this review, we will mainly focus on introducing GLP-1R agonists and trials, and briefly describe the latest research progress of double or triple-target drugs. We anticipate that this study will help biomedical researchers or clinical workers to treat obesity by providing ideas for developing novel drug strategies or scientific research ideas.

## Author contributions

J-YW and Q-WW wrote the first draft of the manuscript. D-RL, J-YJ, and H-CZ revised the manuscript. WY and X-YY made the figures and table. X-FZ critically revised the manuscript. All authors contributed to the article and approved the submitted version.
